# Dynamic Changes of EKG by Severe Hyperkalemia: Transient Left Bundle Branch Block

**DOI:** 10.7759/cureus.36124

**Published:** 2023-03-14

**Authors:** Taruna Chandok, Somin Lee, Nisha Ali, Amandeep Singh, Sandhya Cautha

**Affiliations:** 1 Internal Medicine, Bronx Care Health System, Bronx, USA; 2 Internal Medicine, BronxCare Hospital Center, Icahn School of Medicine at Mt. Sinai, New York City, USA; 3 Cardiovascular Medicine, Bronx Care Health System, Bronx, USA

**Keywords:** rhabdomyolysis, chronic kidney disease, ekg changes, hyperkalemia, left bundle branch block

## Abstract

Hyperkalemia is one of the most common electrolyte abnormalities seen in clinical practice and the most common life-threatening electrolyte abnormality seen in the emergency department. It is most often due to impaired renal potassium excretion due to acute on chronic kidney disease or the use of drugs that inhibit the renin-angiotensin-aldosterone axis. The most common clinical presentation is muscle weakness and cardiac conduction abnormalities. In the Emergency Department, ECG can come in handy as the first diagnosis of hyperkalemia before labs are drawn and reported. Early recognition of electrocardiographic (ECG) changes can prompt early correction and reduce mortality. We hereby, present a case of transient left bundle branch block in the setting of hyperkalemia secondary to statin-induced rhabdomyolysis.

## Introduction

The left bundle branch block (LBBB) is an intraventricular conduction defect that was first reported by Lewis in 1913 [1]. Though a transient bundle branch block is regarded as a precursor for permanent conduction defect, its prognosis is better than a permanent bundle branch block [2,3]. Association of LBBB with hyperkalemia is a rare and uncommon occurrence and has scarcely been reported in the literature. We present a rare association of transient LBBB seen with hyperkalemia in patients presenting with chest pain. Laboratory workup revealed rhabdomyolysis resulting in severe hyperkalemia and acute on chronic kidney injury. This acute presentation of hyperkalemia in the setting of acute kidney injury (AKI) resulted in electrocardiographic changes. These changes seen on ECG manifested as transient LBBB with a return to normal conduction on hemodialysis.

## Case presentation

A 79-year-old female presented to the emergency department with a chief complaint of chest pain. The chest pain was retrosternal, non-exertional, and pressure-like. It was associated with mild shortness of breath and palpitations. Chest pain lasted about one hour and resolved on its own. The review of the system was unremarkable except for decreased urine output for the last two days. On arrival, the patient's vital signs were stable. Blood pressure was 143/61 mmHg, pulse 94 bpm, temperature 97.6 degrees F, and pulse oximeter showed 98% oxygen on room air. The patient was comfortable and not in distress. Physical examination was unremarkable. Cardiac examination showed normal S1, and S2 with no murmur, gallop, or rubs.

The patient had frequent visits to the emergency department in the past for atypical angina. Her medical condition includes coronary artery disease with drug-eluting-stent (DES) in mid-left anterior descending (LAD) and left circumflex (LCX) arteries, peripheral vascular disease, breast cancer in remission, uncontrolled hypertension, uncontrolled type 2 diabetes mellitus, hyperlipidemia and stage IV chronic kidney disease. She was on atorvastatin 40mg, ezetimibe 10 mg, clopidogrel 75 mg, Sitagliptin 25 mg, insulin glargine 12 units at bedtime, isosorbide mononitrate 10 mg, losartan 50 mg, aspirin 81 mg, multivitamins and folic acid.

**Figure 1:**
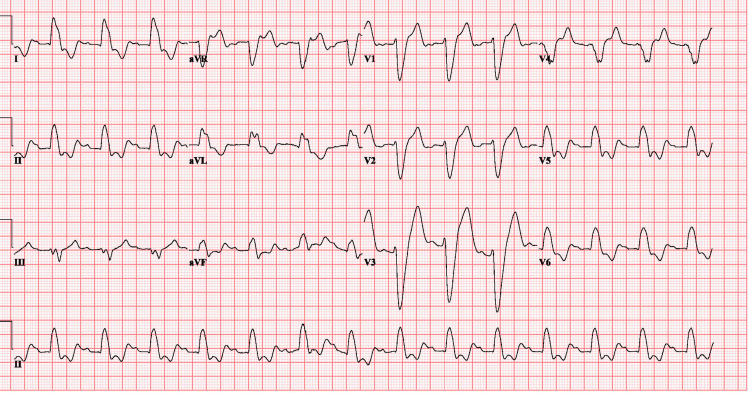
ECG showing left bundle branch block

The patient's initial ECG (Figure 1) on arrival showed normal sinus rhythm with a heart rate of 86, PR interval of 196 ms, wide QRS complex with a duration of 216 ms with dominant deep S-wave in V1, and broad monomorphic R-wave in V6, suggestive of complete LBBB. Compared to the patient's prior baseline ECG, this was a new onset LBBB.

Laboratory results (Table 1) showed severe hyperkalemia with serum potassium (K) of 9.3mEq/L and AKI with creatinine of 8.6 mg/dL (baseline creatinine was 2.1-2.7), blood urea nitrogen (BUN) 117 mg/dL, serum sodium 137 mEq/L, chloride 100 mEq/L, bicarbonate 9 mEq/L, calcium 9.6, magnesium 2.2 mEq/L, phosphorus 8.2 mEq/L and transaminases with aspartate transaminase (AST) 773 u/L and alanine transaminase (ALT) 376 u/L. 

The patient's AKI and hyperkalemia resulted from rhabdomyolysis. The cause of this was attributed to the increase in the dose of statin to 40 mg recently two months back. (The patient was on atorvastatin 10 mg previously for the past one year.) This resulted in elevated serum creatinine kinase level of 24,779 u/L. Urine analysis showed large blood but few RBCs. Laboratory data also showed elevated high sensitivity troponin 582 ng/L, which seems to be laboratory cross-reactivity with serum creatinine kinase and from decreased renal clearance from AKI. The patient also underwent transthoracic echocardiography in the setting of chest pain, rising troponin and new onset LBBB which showed ejection fraction of 63%, concentric left ventricular hypertrophy, no wall motion abnormality, trace mitral and tricuspid regurgitation, insufficient tricuspid regurgitation to assess right ventricular systolic pressure and no pericardial effusion.

**Table 1:** Laboratory results at the time of admission and post hemodialysis HD- Hemodialysis

Labs	On Admission		Day 1 After HD	Day 2 After HD	Reference Range
Time	00.27	12:54	18:34		
Sodium	132	137	138	139	135-145 mEq/L
Potassium	8.5	9.3	5.0	4.5	3.5-5.0 mEq/L
Bicarbonate	13	9	26	25	24-30 mEq/L
Blood Urea Nitrogen	99	117	47	50	6.0-20.0 mg/dL
Creatinine	7.9	8.6	4.1	4.8	0.5-1.5 mg/dL
Chloride	98	100	100	105	98-108 mEq/L
Calcium	9.4	9.6	7.9	8.0	8.5-10.5 mg/dL
Magnesium		2.6	2.0	2.0	1.5-2.7 mg/dL
Phosphorus		8.2	5.1	5.1	2.5-4.5 mg/dL
Aspartate transaminase		773	355	331	9-36 unit/L
Alanine transaminase		376	283	303	5-40 unit/L
Creatinine Kinase	>20,000	24,779		4,032	20-200 unit/L
Troponin	582	610			=<12 ng/L

The patient received calcium gluconate, albuterol inhalation, lokelma (sodium zirconium cyclosilicate), and intravenous insulin regular with dextrose for hyperkalemia management. However, the patient's hyperkalemia was resistant to potassium-lowering medical treatment and hence, she underwent urgent hemodialysis.

**Figure 2:**
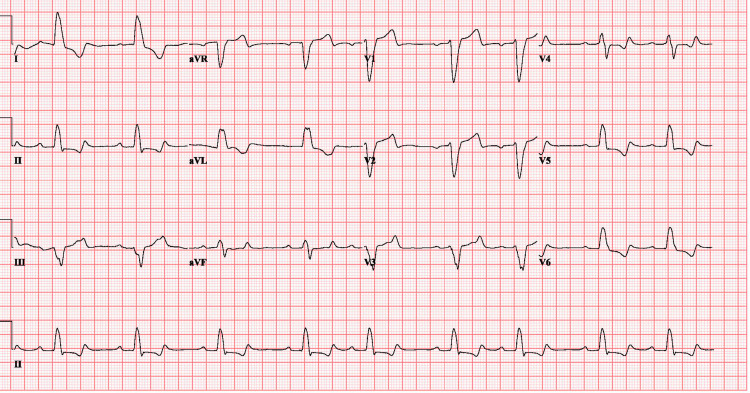
ECG changes after hemodialysis

After hemodialysis, the patient's potassium trended down to 5.0mEq/L. The repeat ECG (Figure 2) a couple of hours after hemodialysis showed dynamic changes in LBBB, PR interval shortened to 154 ms, still wide but narrower QRS complex with a duration of 146 ms compared to initial EKG, dominant S-wave is still present in V1, but the peaked T-wave improved as compared to the initial ECG.

**Figure 3:**
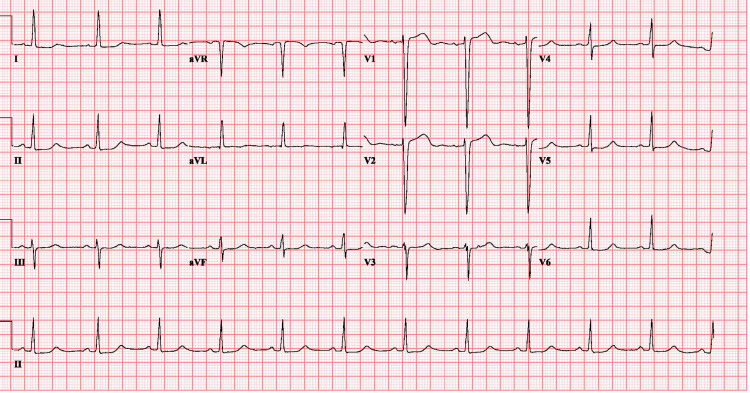
ECG a day after showing normal sinus rhythm

The next day, the patient underwent another session of hemodialysis given her persistent hyperkalemia, K of 5.9 mEq/L. After K improved to 4.5 mEq/L. ECG (Figure 3) showed normal sinus rhythm with resolved LBBB.

AKI was from rhabdomyolysis and the etiology of rhabdomyolysis was suspected statin-induced myopathy. The patient had transaminitis likely due to statin use at home. The patient received intermittent hemodialysis until her kidney function improved and rhabdomyolysis resolved and was discharged home.

## Discussion

With the advent of the treatment for hyperlipidemia, statins have grown in use since 1987. With their increased use comes their adverse effects, statin-induced myopathy is one of them and its incidence is 1.5 million people per year [4]. The clinical presentation of statin-induced myopathy includes myalgias, myopathy, rhabdomyolysis, and an asymptomatic increase in creatine kinase. Rhabdomyolysis results in skeletal muscle breakdown resulting in the release of intracellular contents into the bloodstream. This causes electrolyte abnormalities (hyperkalemia), myoglobinuria, and an increase in creatine kinase levels. Myoglobin has been implicated as a dominant cause of rhabdomyolysis-induced acute renal failure. Myoglobin exerts its nephrotoxic effects by inducing renal vasoconstriction and ischemia, direct cytotoxic effects, and cast formation in the distal convoluted tubules. Potassium is largely stored intracellularly. The intracellular potassium concentration is approximately 140 mEq/L compared to 4-5 in the extracellular group. The release of intracellular contents into the bloodstream with rhabdomyolysis can result in life-threatening levels of extracellular potassium. This clinically manifests as muscle weakness or paralysis followed by cardiac conduction abnormalities and cardiac arrhythmias.

An acute increase in potassium levels can result in electrocardiogram (ECG) manifestations [5]. The series of ECG abnormalities include peaking of T waves with shortened QT interval, PR interval prolongation, QRS prolongation, loss of sinoatrial conduction with the onset of a wide-complex “sine-wave” ventricular rhythm, and ultimately asystole. The more severe manifestations occur with serum potassium concentrations >9 mEq/L [6]. The sensitivity of ECG changes in hyperkalemia is uncertain. Only 46% of patients with renal insufficiency presenting with hyperkalemia were noted to have electrocardiographic changes, but no clear criteria were presented in the prospective series of case reports [7].

Interventricular Conduction delay induced by hyperkalemia can either take the form of a right or left bundle branch block. Right bundle brand block can be found in young healthy adults but the incidence of LBBB increases with age. Though commonly associated with conditions causing myocardial fibrosis such as hypertension, coronary artery disease, and cardiomyopathies, intermittent LBBB is a rare occurrence. It has been reported with exercise, tachycardia, ischemic and hypertensive heart disease, hyperkalemia, drugs, anesthesia, pulmonary embolism, chest trauma, and cardiac interventional procedures [8-12]. Apart from transient LBBB, RBBB, bifascicular block, and advanced AV block have also been reported [13-15].

Our patient presented with severe hyperkalemia of 9.3 mEq/L caused by rhabdomyolysis complicated by AKI. The patient’s recent home medications showed the use of statins with transaminitis on laboratory findings which was suspected to be the cause of severe rhabdomyolysis. This severity of hyperkalemia resulted in ECG findings of a new onset LBBB when compared with the patient’s previous ECGs. Given the association of transient LBBB with ischemia in patients with known or suspected coronary artery disease, it can be argued that this could have been a manifestation of myocardial infarction given the chest pain, elevated troponin, and history of extensive coronary artery disease. Though transthoracic echocardiography did not show any wall motion abnormality. The patient also had a cardiac catheterization, a year before this admission which showed non-obstructive CAD with patent stents. CAD was less likely to be the cause. Later, these changes were resolved with hemodialysis and hence found to be transient in nature with normalization of potassium levels. The patient's chest pain was diagnosed as atypical angina with a normal cardiovascular exam, and it resolved on its own.

High potassium levels are associated with transmembrane permeability changes which can be suppressed by intravenous calcium chloride infusion or administration of a combination of insulin and glucose which can transfer the potassium from extracellular compartment to Intracellular compartment. Beta 2 agonists and sodium bicarbonate can also be helpful.

In patients presenting with hyperkalemic emergency defined as severe hyperkalemia (>6.5 mEq/L) and cardiac conduction abnormality observed in our patient, IV calcium, Insulin, and glucose is the rapidly acting therapies. In addition, therapies that remove potassium from the body should be administered such as hemodialysis, gastrointestinal cation exchangers, and diuretics.

Our patient received repeated hemodialysis and was monitored on telemetry. Her kidney function improved, rhabdomyolysis resolved, and was discharged home.

## Conclusions

Transient LBBB is a rare, but important presentation of hyperkalemia seen on ECG. With the growing use of renin-angiotensin inhibitors especially in patients with chronic kidney disease, statin-induced myopathy, and acidotic states, this can be a life-threatening presentation. A thorough knowledge of the ECG manifestations related to hyperkalemia is crucial to ensure emergent treatment. This can improve outcomes in patients with cardiovascular and renal disease.
